# Dr. Allport’s Address

**Published:** 1874-10-15

**Authors:** 


DR. ALLPORT'S ADDRESS.—The annual address before the American Association of Dental Surgeons, at the recent meeting in Boston, was delivered by Dr. W. W. Allport of this city.

The address, aside from being well written and interesting throughout, presented a leading idea which is worthy of a careful consideration. It is, that dentistry as now practiced, embraces two distinct departments: first a scientific part, relating to the pathology and treatment of the natural teeth and their appendages; the second a mechanical part, relating to the manufacture and adjustment of artificial appliances. The first is as much a department of medicine proper as ophthalmology, otology, gynecology, etc., and should be so recognized and taught in the regular medical colleges; while the second should have the same relation to it, that the surgical instrument maker does to the practical surgeon. If the distinction thus clearly indicated in the address, could be generally adopted in practice, and dentology proper reunited with the general field of medicine and surgery, and those who practice it be recognized as specialists in the same sense as other medical specialists, it would doubtless elevate the position and enhance the usefulness of that department of practice.

It would not merely save the expense of separate colleges of dentistry, but the recognition of dental pathology and therapeutics as a part of medical science; and the establishment of a chair for teaching these branches in the regular medical colleges, would result in giving the dentologist something of a general medical education on the one hand, and the general practitioner a better knowledge of dental science on the other. The usefulness of both parties would be thereby increased.

